# First-line chemotherapy for ovarian cancer – the controversy continues

**DOI:** 10.1038/sj.bjc.6600568

**Published:** 2002-10-07

**Authors:** S B Kaye

**Affiliations:** The Royal Marsden Hospital/Institute of Cancer Research, Downs Road, Sutton, Surrey, UK

## Abstract

*British Journal of Cancer* (2002) **87**, 813–814. doi:10.1038/sj.bjc.6600568 www.bjcancer.com

© 2002 Cancer Research UK

## 

The choice of optimal first-line chemotherapy for advanced ovarian cancer continues to excite controversy. In the late 1990s the widespread recognition that carboplatin represented a less toxic and equally efficacious alternative to cisplatin was a major step forward. At the same time, the introduction of paclitaxel appeared to be a significant advance, based on a survival benefit in two large randomised trials, i.e. GOG1-11 and OVO-10 (Mcguire *et al*, 1996; Piccart *et al*, 2000). Funding difficulties were eventually resolved in the UK by a positive NICE assessment 2 years ago, confirming the role of paclitaxel in first-line combination therapy.

Since then, however, doubts have arisen, largely over two randomised trials, GOG 132 and ICON 3, which essentially indicate that single agent platinum therapy is as effective as paclitaxel-platinum combination. These seemingly conflicting data are well described and discussed in the review in this journal issue, by Sandercock *et al*. They propose that the likeliest explanation of these data is that the control arms differed substantially; cyclophosphamide-cisplatin being less effective than the other options. They conclude that ‘single agent carboplatin is a safe and effective first-line treatment for women with advanced ovarian cancer.’

The data as described would be compatible with the notion that cyclophosphamide-cisplatin represented a suboptimal control arm; nevertheless it did represent standard treatment in many centres until the mid 1990s and this was based on other trials showing the benefit compared to single agent platinum. If the authors are correct in their assertion, some further consideration of this explanation would have been helpful. Biochemical antagonism between cyclophosphamide and platinum at the tumour cell itself has been suggested although seems unlikely, but it is conceivable that some aspect of the platinum dose is relevant. In both GOG 1-11 and OVO-10, cyclophosphamide-induced toxicity led to a greater number of delays in treatment in that arm, compared to the paclitaxel arms, resulting in significantly lower cisplatin dose-intensities actually received. Whilst reviews of various trials indicate that increasing dose-intensity for platinum has doubtful benefit (Vasey *et al*, 1998), it is possible that reductions below an optimal threshold could well lead to an inferior result.

Although it is hard to contradict the authors' concluding statement, those of us involved in managing this disease clearly need to go forwards rather than backwards from here. Further detailed comparisons of these four studies done at different times by different groups using different treatments can be useful in formulating hypotheses, but any conclusions should be drawn with caution, not least because of the heterogeneity of treated populations. Taxanes should remain an important element in the first line of management of these patients, for several reasons. There is clear evidence of efficacy in patients whose disease no longer responds to platinum, implying that the target cancer cell population is somewhat different, and this is supported by experimental data focusing on the presence or absence of functional p53 (Wahl *et al*, 1996). In addition, the mechanism of action and the toxicity profiles are quite different from platinum. For virtually all chemosensitive epithelial cancers, combination chemotherapy rather than single agent treatment has provided significant survival benefits when applied as initial therapy, and there seems no fundamental reason for concluding that ovarian cancer will ultimately prove to be different in this regard. The challenge now remains: how to identify the optimal combination?

The two trials, GOG 132 and ICON 3, do suggest that paclitaxel-cisplatin or paclitaxel-carboplatin, given as concurrent treatments every 3 weeks for 6 cycles with a 24 h or three paclitaxel infusion may not necessarily be the best approach. It is conceivable, indeed, that the two drugs exhibit a degree of antagonism in certain schedules, and experimental data on this are equivocal.

Alternative approaches should clearly be considered for clinical trial research and these could include sequential regimes, in which the taxanes and the platinum compound are given separately. The impact of modulators of response to chemotherapy, such as EGFR tyrosine kinase inhibitors, also merits serious consideration, since they may have an important effect on any taxane-platinum interactions. These and other approaches, such as the further evaluation of ‘maintenance chemotherapy’ with single agent paclitaxel, and the incorporation of other active agents such as liposomal doxorubicin, topotecan and gemcitabine provide a rich seam for clinical triallists in the next few years.

Meantime, the management of the individual patient outside a clinical trial will continue to excite debate. Although clinical practices are not uniform, it is probably true to say that for some clinicians the threshold for withholding paclitaxel from first line therapy may have changed as a result of GOG 132 and ICON 3. As pointed out by Sandercock *et al*, a pooling of the overall data utilising the random effects model does suggest a benefit for paclitaxel-platinum of borderline significance (hazard ratio for progression free survival of 0.84, 95% confidence intervals of 0.70 and 1.01, *P*=0.06). At this time it is therefore reasonable to continue to offer paclitaxel along with carboplatin to appropriate patients as first-line therapy, using schedules in widespread usage. This should be accompanied by a careful explanation of the relatively modest likely benefit in addition to carboplatin. More important still will be the entry of such patients into appropriate first line chemotherapy studies. These should aim to address issues such as those described above, so that these dilemmas in management can be resolved as soon as possible. The thousands of patients who have taken part in the trials described, and their families, deserve nothing less.
